# Fast and Sensitive HPLC-ESI-MS/MS Method for Etoricoxib Quantification in Human Plasma and Application to Bioequivalence Study

**DOI:** 10.3390/molecules27175706

**Published:** 2022-09-04

**Authors:** Gabriel Onn Kit Loh, Emily Yii Ling Wong, Yvonne Tze Fung Tan, Siew Chyee Heng, Mardiana Saaid, Kit Yee Cheah, Nurul Diyana Mohd Sali, Nair Damenthi, Sharon Shi Min Ng, Long Chiau Ming, Kok Khiang Peh

**Affiliations:** 1 Bioxis Sdn. Bhd. PMT 1241, Jalan Perindustrian Bukit Minyak 8, Taman Perindustrian Bukit Minyak, Simpang Ampat 14100, Malaysia; 2 School of Chemical Sciences, Universiti Sains Malaysia, Minden 11800, Malaysia; 3 Centre for Clinical Trial, Institute for Clinical Research, National Institutes of Health, Ministry of Health Malaysia, Hospital Ampang, Ampang 68000, Malaysia; 4 PAP Rashidah Sa’adatul Bolkiah Institute of Health Sciences, Universiti Brunei Darussalam, Gadong BE 1410, Brunei; 5 School of Pharmaceutical Sciences, Universiti Sains Malaysia, Minden 11800, Malaysia

**Keywords:** etoricoxib, etoricoxib D4, protein precipitation, high-sample throughput, human plasma, bioequivalence study

## Abstract

Etoricoxib is a non-steroidal anti-inflammatory drug (NSAID) used to treat pain and inflammation. The objective of the current study was to develop a sensitive, fast and high-throughput HPLC-ESI-MS/MS method to measure etoricoxib levels in human plasma using a one-step methanol protein precipitation technique. A tandem mass spectrometer equipped with an electrospray ionization (ESI) source operated in a positive mode and multiple reaction monitoring (MRM) were used for data collection. The quantitative MRM transition ions were *m/z* 359.15 > 279.10 and *m/z* 363.10 > 282.10 for etoricoxib and IS. The linear range was from 10.00 to 4000.39 ng/mL and the validation parameters were within the acceptance limits of the European Medicine Agency (EMA) and Food and Drug Analysis (FDA) guidelines. The present method was sensitive (10.00 ng/mL with S/N > 40), simple, selective (K prime > 2), and fast (short run time of 2 min), with negligible matrix effect and consistent recovery, suitable for high throughput analysis. The method was used to quantitate etoricoxib plasma concentrations in a bioequivalence study of two 120 mg etoricoxib formulations. Incurred sample reanalysis results further supported that the method was robust and reproducible.

## 1. Introduction

Pain has been reported as one of the current common health issues in the population [1]. According to the International Association for the Study of Pain, about 10% of adults are diagnosed with chronic pain every year [2]. People suffering from moderate to severe chronic pain find it difficult to maintain their daily lifestyles. Studies also reveal that pain causes strained or broken relationships with friends and family [3]. Certain pain, such as nociceptive pain, is often linked with inflammation due to the stimulation of unaltered nociceptors by external stimuli and release of substances causing the pain [4]. Non-steroidal anti-inflammatory drugs (NSAIDS), which have analgesic, antipyretic and anti-inflammatory effects, have been widely used for the treatment of pain [5,6,7,8].

**Figure 1:**
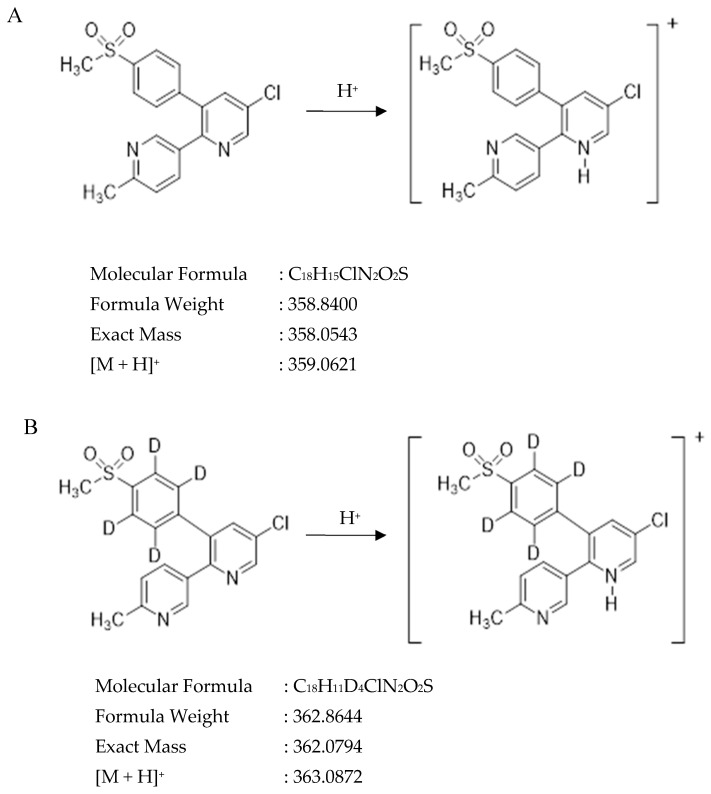
Molecular structures of (**A**) etoricoxib and (**B**) etoricoxib D4.

Etoricoxib, chemically known as 5-chloro-6′-methyl-3-[4-(methylsulfonyl)phenyl]-2,3′-bipyridine, is an oxicam class NSAID. It is a Class II drug under the Biopharmaceutical Classification System (BCS) with low solubility and high permeability [9,10,11]. Etoricoxib is a cyclooxygenase (COX)-2-selective inhibitor used in the treatment of pain and inflammation in osteoarthritis, rheumatoid arthritis, acute gouty arthritis, dental surgery, gout and chronic pelvic pain [12,13,14] due to its antipyretic, analgesic and anti-inflammatory effects. Etoricoxib shows a lower risk of gastrointestinal toxicity and comparable efficacy to traditional NSAIDs in patients with osteoarthritis. Therefore, etoricoxib has greater potential to be used as pain relief medicine in the elderly population [15]. Figure 1 shows the molecular structures of etoricoxib and etoricoxib D4.

Etoricoxib is well absorbed after oral administration (approximately 83% absolute bioavailability). After intravenous (i.v.) and oral administration (p.o.) doses, about 75% of etoricoxib accounts for the majority of the radioactivity present in plasma [13]. The maximum plasma concentrations (C_max_) are 95–2186 ng/mL after administration of 5–120 mg of etoricoxib and the time to achieve C_max_ is 1 h [16]. The excretion is mainly through the hepatic route and its half-life is approximately 22 h. Etoricoxib exhibits a linear pharmacokinetics over the clinical dose range of 30–240 mg [16,17]. More than 90% of etoricoxib is metabolized and excreted through urine and feces. Only 1% of the dose is excreted in the urine as unchanged drug. There are several enzymes responsible for the metabolism of etoricoxib in liver, e.g., CYP3A4, CYP2C9, CYP1A2, CYP2C19 and CYP2D6 [9,10,18,19].

A fast, sensitive and high throughput bioanalytical method is desired for the analysis of studies involving a high number of plasma samples. Analytical methods using high performance chromatography (HPLC) coupled with ultraviolet detector [20,21,22,23,24,25] and tandem mass spectrometer [26,27,28,29] have been reported for estimation of etoricoxib in human plasma as reported in Table 1. The limitation of the HPLC-UV method is the lack of sensitivity, use of large injection volume and a long sample analysis time. Sample preparation is either solid phase or liquid–liquid extraction to improve the detection sensitivity, which is tedious and time consuming. The use of a more sensitive HPLC-MS/MS method is anticipated to circumvent the limitations of the HPLC-UV methods. Solid phase extraction (SPE) was employed by Bräutigam et al. [26] while liquid–liquid extraction (LLE) was used by Werner et al. [28] and Junior et al. [30] in sample preparation to achieve sensitivity of 10 ng/mL and 1 ng/mL with 20 μL of injection volume. Zhang et al. [29] reported a protein precipitation technique, but there was no information on system suitability, re-injection reproducibility, extended batch-run precision and accuracy or incurred sample reanalysis (ISR).

The objective of the present study was to develop a fast and sensitive HPLC-ESI-MS/MS method to quantify etoricoxib in human plasma using a one-step methanol protein precipitation technique. The use of methanol as a protein precipitation agent demonstrated comparatively higher sensitivity than that of acetonitrile. A small injection volume of 1 μL could minimize the contamination of the ESI interface, reducing the downtime and maintenance frequency of the instrument. Furthermore, a short sample analysis time of 2.0 min at a flow rate of 400 μL/min minimized the generation of non-environmentally friendly organic solvent waste. The validated etoricoxib method was applied to a bioequivalence study of two formulations of etoricoxib and incurred sample reanalysis (ISR).

## 2. Results and Discussion

### 2.1. Method Development and Optimization

Etoricoxib has a pKa value of around 4.6 and it is categorized as a weak basic drug [32]. The molecular structure of etoricoxib has four hydrogen bond acceptor counts with no hydrogen bond donor count. Therefore, etoricoxib is easily protonated to form positively charged molecular ions. Etoricoxib and etoricoxib D4 (IS) full scan positive modes showed the predominant protonated molecular ions [M + H]^+^ at 359.15 and 363.10. Only two fragment peaks with the highest signal intensity were selected as quantification and confirmation product ions for etoricoxib and IS after performing MRM optimization. The selected quantitative and confirmation product ions were *m/z* 359.15 > 279.10 and 359.15 > 280.10 for etoricoxib and *m/z* 363.10 > 282.10 and 363.10 > 284.15 for IS. Q1 pre bias collision and Q3 pre bias energies during MRM optimization for mass transition of etoricoxib and IS are shown in Appendix A (Table A1).

Chromatographic conditions such as type of column, mobile phase composition, sample injection volume and flow rate were optimized to improve the sensitivity (S/N), symmetry factor (tailing factor) and capacity factor (K prime) for etoricoxib and IS. Poroshell 120 EC-C18 and Kinetex XB-C18 columns exhibited a better symmetry factor than Shim-Pack Velox C18 column. Poroshell 120 EC-C18 and Kinetex XB-C18 gave better integration of the peak response for both etoricoxib and IS. In terms of sensitivity, the two analytical columns, Poroshell 120 EC-C18 and Kinetex XB-C18, reported S/N ratio values of more than 30 at 10.00 ng/mL, which was the LLOQ of the study. Based on the dimension and interstitial porosity, the estimated void times of Poroshell EC-C18 and Kinetex XB-C18 columns were 0.43 and 0.30 min, with a flow rate of 400 μL/min. The calculated capacity factors were 1.53 and 2.63 for Poroshell EC-C18 and Kinetex XB-C18 columns, at a retention time of 1.09 min for both analyte and IS as both have similar chemical properties. K prime is very important as a means of measuring the retention of analyte and IS in the column. K prime value should be more than 1 to avoid the analyte and IS eluting with other endogenous components in the samples. The Kinetex XB-C18 column was selected in this study due to its higher capacity factor than Poroshell EC-C18 column, giving better retention for both analyte and IS.

Methanol was used as an organic solvent in the mobile phase because methanol gave five times higher sensitivity than acetonitrile for both etoricoxib and IS. The percentage of formic acid in mobile phase which acted as proton donor was found to affect the sensitivity of both analyte and IS. The percentage of formic acid was optimized from 0.01 to 0.1% and both analyte and IS showed the highest sensitivity with 0.05% formic acid in the mobile phase. A flow rate of 400 μL/min with a mobile phase composition of 0.05% formic acid and methanol (2:3, *v/v*) was the most optimal chromatographic system with good separation, no interference peaks and retention time of 1.09 min for both analyte and IS. The run time of 2.0 min enabled a high throughput sample analysis of etoricoxib in a bioequivalence study. In addition, the use of a low flow rate also reduced the consumption of organic solvents and overall cost of analysis. Based on the published C_max_ values of etoricoxib in different subject populations after administration of 120 mg etoricoxib under fasting conditions, ranging between 1923.90 to 2364.78 ng/mL, the LLOQ value of ≤ 95 ng/mL should be achieved to fulfil the requirement of at least 5% of C_max_ value in a bioequivalence study [29,31].

The protein precipitation technique was used in this study due to its simplicity and fewer processing steps [33]. Furthermore, methanol was found to exhibit a higher extraction efficiency than acetonitrile as protein precipitation agent. The extraction efficiency was >92% for methanol and <50% for acetonitrile at a mobile phase composition of 0.05% formic acid and methanol (2:3, *v/v*). Ratio of 1:4 (plasma:methanol, *v/v*) produced a clean sample without any matrix effect. Matrix factors of 1.06 and 1.01 were obtained for low QC (QC_L_) and high QC (QC_H_).

SPE and LLE methods do not support high-throughput etoricoxib sample analysis as these techniques involve multiple steps, encompassing pipetting, vortex mixing, centrifuging, organic solvent transferring, drying and reconstituting, which are time consuming [21,22,23,24,25,26,27,28,30,31]. The calibration range reported by published studies might not be sufficient to cover the etoricoxib concentration in the human plasma samples collected over a predetermined sampling time where a number of plasma sample concentrations might fall outside the studied calibration range [20,25,26]. Zhang et al. [29] employed an acetonitrile protein precipitation technique, but the method employed a double 96-well plate in sample preparation which increased the cost of analysis. A gradient method with a flow rate of 0.6 mL/min and analysis time of 2.5 min used in the study consumed 87.5% more of mobile phase than that of our method. Our proposed method could overcome the limitations of the published methods.

### 2.2. System Suitability

A coefficient of variation (CV, %) of ≤3.43 for 3250.32 ng/mL system suitability samples at the initial run showed that the mass system reached equilibration before the start of the bioanalytical method validation. A deviation percentage of ≤14.65% for system suitability samples throughout the run was reported. No carry over effect was detected for both analyte and IS in the system suitability test.

### 2.3. Method Validation

**Figure 2:**
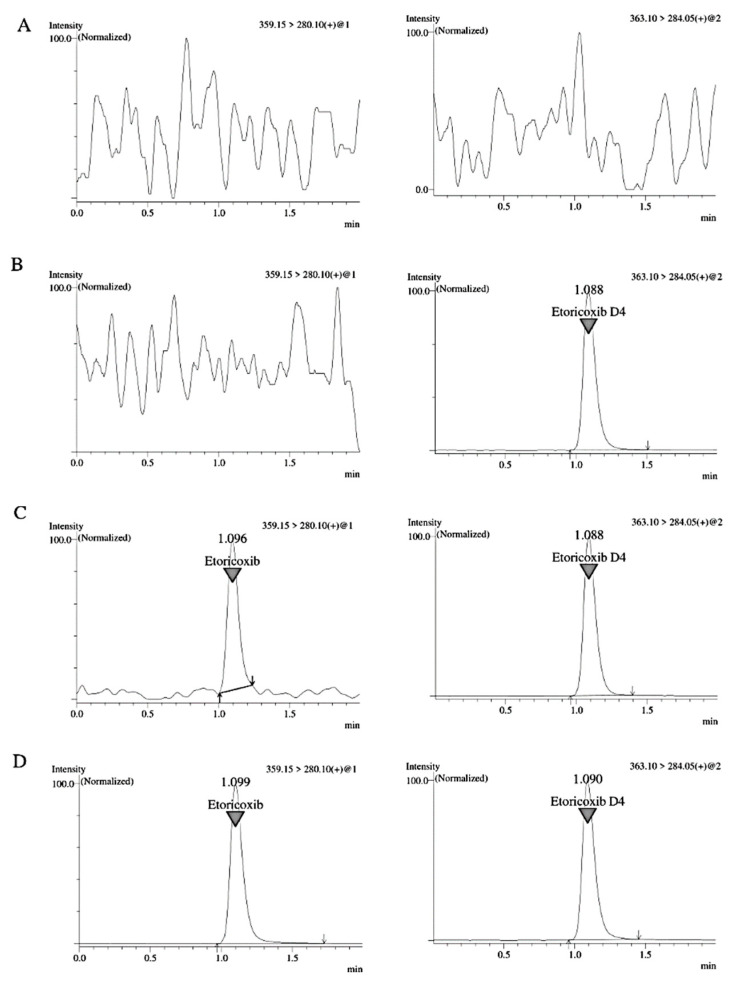
Mass chromatograms of (**A**) blank sample, (**B**) zero sample, (**C**) LLOQ of etoricoxib (10.00 ng/mL) and (**D**) a subject’s sample at 2400.45 ng/mL concentration.

No interference was found at the analyte and IS retention times for seven batches of blank human plasma. Figure 2 illustrates the mass chromatograms of blank, zero sample, LLOQ and one subject sample (2400.45 ng/mL etoricoxib). Zero sample also indicated the absence of interfering peaks at the etoricoxib retention time. The mean S/N ratios of analyte and IS were greater than 40 and 4000, showing that the method was highly sensitive for both analyte and IS at a retention time range of 1.08–1.10 min.

Etoricoxib plasma calibration curves were linear with correlation of determinations (r^2^) of 0.9967–0.9996 over concentration range of 10.00 to 4000.39 ng/mL. The relative error (RE, %) for all the calibration points was ± 10.89% of their nominal concentration which was within the acceptance limit. Carry-over was not found in the blank human plasma sample which was injected after ULOQ sample.

The within- and between-run precision and accuracy results are shown in Table 2. The precision (CV, %) and within-run accuracy (bias, %) of QC samples (including LLOQ) ranged from 0.64% to 16.67% and −4.19% to 7.04%. Between-run precision (CV, %) and accuracy (bias, %) values ranged from 2.25% to 13.74% and −1.68% to 5.86%, respectively. The etoricoxib maximum batch-run precision (CV, %) and accuracy (bias, %) results of ≤6.75% and ≤8.77% showed that the method would be suitable for an analytical batch-run with maximum run time of 250 min. The precision and accuracy results show that the method is precise and accurate.

The mean IS normalized matrix factors for etoricoxib were 1.04 ± 0.06 and 1.06 ± 0.01 for QC_L_ and QC_H_ levels, respectively, with CV (%) < 5.93%, indicating negligible matrix effect and no ion enhancement or suppression. The mean recovery values of etoricoxib and IS are shown in Table 3. The extraction recovery of analyte and IS was greater than 91% with a CV (%) < 7.73%.

The accuracy (bias, %) values of 2-fold and 10-fold dilutions were 0.23% and 5.29%, while the precision (CV, %) values were 1.10% and 0.46% (Table 4), respectively.

The deviation percentage (%) between initial and re-injected QC samples was less than 9.10%. Initial and re-injected plasma calibration curves reported r^2^ values of more than 0.99 and the relative errors (RE, %) for all the calibration points were within ±5.86 from their nominal concentration. These results indicate that the method is highly reproducible.

The stability study results of etoricoxib in different testing conditions are presented in Table 5. Etoricoxib was stable when stored at room temperature for 24 h, after seven freeze–thaw cycles and 94 days at −20 ± 10 °C in human plasma. The processed etoricoxib plasma samples were stable at room temperature and autosampler for 48 h. Both stock and working standard solutions of etoricoxib and IS were stable at room temperature and in the chiller for 94 days. The stability results showed that no degradation of etoricoxib was observed during the sample analysis time.

### 2.4. Comparative Dissolution Profiles

**Figure 3:**
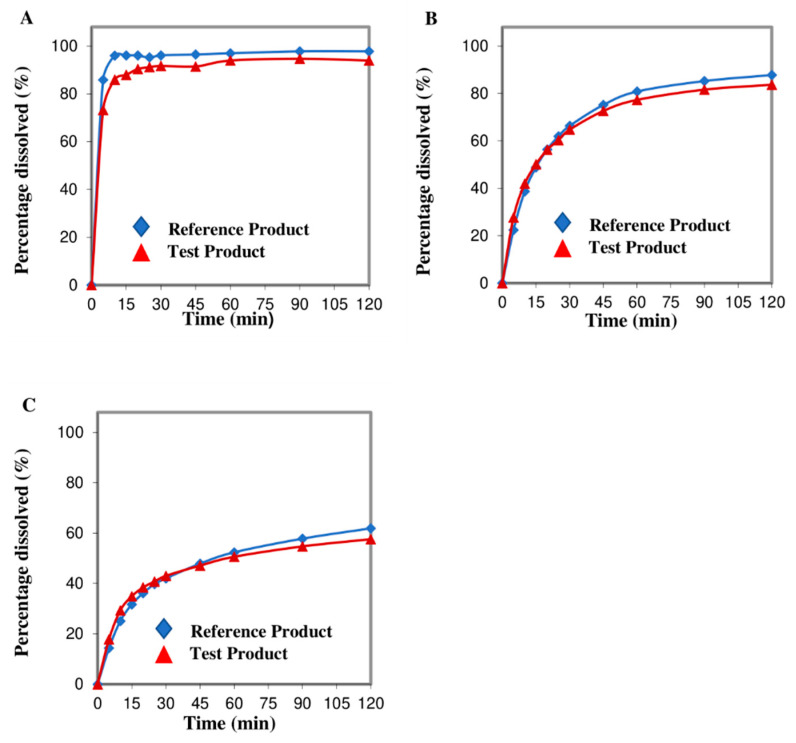
Comparative dissolution profiles between test and reference products for etoricoxib 120 mg at (**A**) pH 1.2, (**B**) pH 4.5, and (**C**) pH 6.8 dissolution medium.

Figure 3 shows the CDP of the two etoricoxib formulations in three different dissolution media. Etoricoxib is a weak basic drug and its dissolution in acidic medium (pH 1.2 and 4.5) was relatively higher than in pH 6.8. More than 85% of etoricoxib dissolved within 15 min for both test and reference formulations at pH 1.2. Therefore, *f*_2_ value was not calculated at pH 1.2. At pH 4.5, more than 80% of etoricoxib released from both formulations at 120 min. At pH 6.8, less than 65% etoricoxib was released from both formulations at 120 min. The *f_2_* values were 74.51 and 76.22 for pH 4.5 and 6.8, showing that both formulations were similar. The test product could proceed with the pivotal bioequivalence study.

### 2.5. Bioequivalence Study

**Figure 4:**
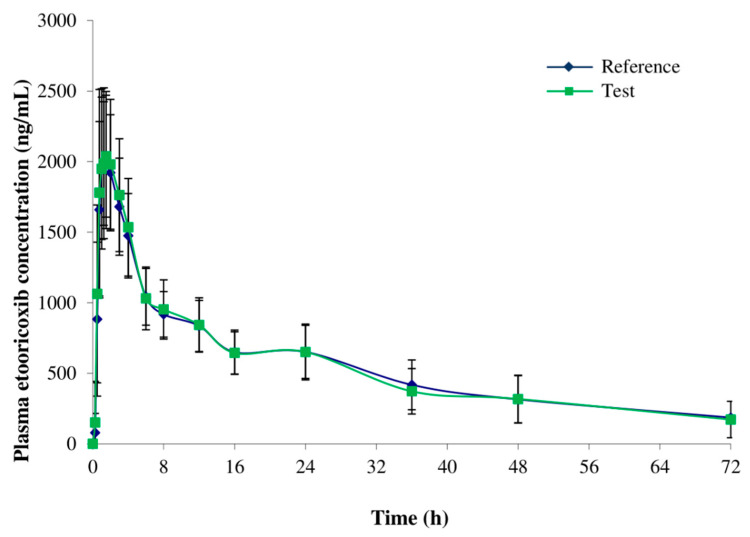
The mean plasma etoricoxib concentration–time profiles of test and reference products of etoricoxib 120 mg.

A total of 18 subjects completed the study. The mean plasma concentration–time curves of etoricoxib are shown in Figure 4. The pharmacokinetic results of etoricoxib are summarized in Table 6. The present bioanalytical method was sufficiently sensitive based on the mean C_max_ values of both test and reference formulations. Furthermore, more than 98% of studied samples were quantifiable and fell within the range of the standard curve (10.00–4000.39 ng/mL). The present LLOQ level was 0.45% of the mean C_max_ values (far below 5% as stated in the guidelines).

The values of the pharmacokinetic parameters of the present bioequivalence study were comparable with the pharmacokinetic results of previously published bioequivalence studies under fasting conditions [21,29,31]. Najib et al. [31] reported C_max_, AUC_0–72_ and t_max_ of 1923.90 ± 466.83 ng/mL, 23,067.3 ± 8978.36 h.ng/mL and 1.34 ± 0.65 h, respectively, for the test product, and 1986.14 ± 614.41 ng/mL, 23,478.2 ± 9719.32 h.ng/mL and 1.26 ± 0.77 h, respectively, for reference product. Tjandrawinata et al. [21] reported C_max_ values of 3155.93 ± 752.81 and 2915.13 ± 772.81 ng/mL, AUC_0–72_ values of 45,913.42 ± 13,142.19 and 44,577.20 ± 13,541.85 h.ng/mL, t_max_ values of 1 and 1 h, for test and reference products, respectively, in Indonesian subjects. Zhang et al. [29] reported C_max_, AUC_0–120_ and t_max_ values of 2364.78 ± 538.01, 44,605.53 ± 15,266.66 h.ng/mL and 2 h, respectively, in Chinese healthy subjects.

**Figure 5:**
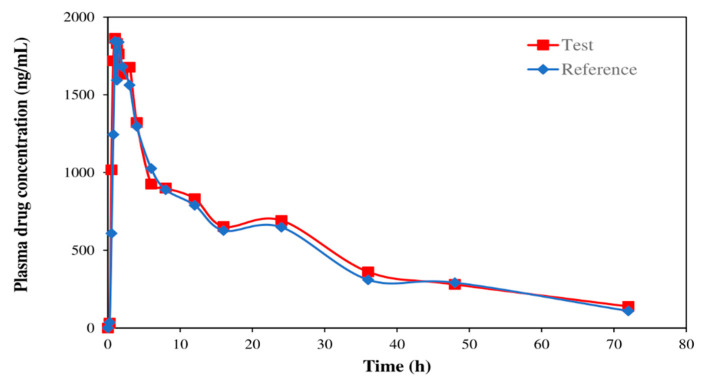
The etoricoxib pharmacokinetic profile of test and reference product of etoricoxib 120 mg for a volunteer.

Individual and mean etoricoxib plasma concentration–time curves (Figure 5) revealed twin peaks consistent with findings of the previously published studies [17,21,34,35]. The secondary peaks could be due to distribution and recirculation from organ compartment back into central blood compartment or enterohepatic recycling [35]. However, the mechanisms contributing to these multiple secondary peaks were unknown [17].

The ln-transformed AUC_0–72_ and C_max_ values between test and reference products showed no statistically significant difference. Similarly, there was no statistically significant difference in the t_max_ values of both products. The 90% confidence interval (CI) for ln-transformed C_max_ and AUC_0–72_ of test over reference products fell within the bioequivalence acceptance limits of 0.80–1.25, showing that the test product is bioequivalent to the reference product. Furthermore, no serious adverse events were found throughout the study. This indicates that the test and reference products could be used interchangeably.

ISR is the repeated measurement of etoricoxib concentration in study samples to demonstrate reproducibility of the method. This study reported an ISR of 98.86%, proving that the developed etoricoxib bioanalytical method is highly robust and accurate.

## 3. Materials and Methods

### 3.1. Chemicals and Reagents

The reference standard of etoricoxib (purity: 99.85%) was supplied by Y.S.P Industries (Kajang, Selangor, Malaysia) while etoricoxib D4 (internal standard, IS, purity: 98.49%) was purchased from Simson Pharma Limited (Mumbai, India). Methanol (LCMS grade) was purchased from J.T. Baker (Deventer, The Netherlands). Formic acid with a purity of >98% was procured from Merck (Darmstadt, Germany). Blank human plasma with dipotassium ethylenediaminetetraacetic acid (K_2_EDTA) anticoagulant was purchased from i-DNA Biotechnology (M) Sdn. Bhd. (Kuala Lumpur, Malaysia). Purified water was produced by a Thermo Fisher Scientific Barnstead Smart2 Pure 12 Ultrapure water system (Pittsburgh, PA, USA).

### 3.2. Instruments and Chromatographic Conditions

The HPLC-ESI-MS/MS system consisted of a Shimadzu Nexera X2 series UHPLC system (Shimadzu Corporation, Kyoto, Japan), binary pumps, a degasser, an autosampler, a system controller, a column oven and a Shimadzu LCMS-8040 tandem mass spectrometer coupled with an electrospray ionization (ESI) source controlled by LabSolutions software (Version 5.91, Kyoto, Kyoto Prefecture, Japan). Positive multiple reaction monitoring (MRM) mode was used to quantify etoricoxib and IS.

The triple quadrupole mass spectrometry conditions were as follows: interface bias/capillary voltage, 4500 V; detector energy, −2120 V; conversion dynode voltage, 6000 V; heat block temperature, 400 °C; desolvation line (DL) temperature, 250 °C; nitrogen drying gas flow, 15 L/min; nitrogen nebulizing gas flow, 3 L/min, and argon collision gas setting, 230 kPa, respectively. Two multiple reaction monitoring (MRM) transitions, quantification and confirmation transitions were used for determination of etoricoxib and IS. The MRM transition of quantification and confirmation of product ions were *m/z* 359.15 > 279.10 and *m/z* 359.15 > 280.10 for etoricoxib, while *m/z* 363.10 > 282.10 and *m/z* 363.10 > 284.15 for IS.

The chromatographic separation of etoricoxib and etoricoxib D4 was performed on a Phenomenex Kinetex XB—C18 100Å (Phenomenex, Torrance, CA, USA) LC column (100 × 2.1 mm; 2.6 μm particle size) connected to a guard column (Security guard ULTRA Cartridges UHPLC C18, 2.1 mm ID column, Phenomenex, Torrance, California, USA) and maintained at 30 °C. The analyte and IS at 1 μL injection volume were eluted isocratically using a mobile phase with a composition of 0.05% formic acid and methanol (2:3, *v/v*) and run at a flow rate of 400 μL/min. The autosampler temperature was 25 °C.

### 3.3. Stock Standard Solutions, Working Standard Solutions, Calibration Standards and Quality Control (QC) Samples

The stock standard solutions of etoricoxib and IS were prepared separately at 100.0 and 20.0 μg/mL in methanol. Working standard solutions of etoricoxib (100.01–40,003.90 ng/mL) and IS (3000.50 ng/mL) were diluted from stock standard or working standard solutions with water and methanol (1:1, *v/v*).

The calibration curves for plasma were prepared by spiking appropriate working standard solutions in blank plasma sample at concentrations of etoricoxib in the range of 10.00–4000.39 ng/mL. Another QC stock standard solution of etoricoxib was prepared for preparation of QC plasma samples comprising of lower limit of quantification (LLOQ), low QC (QC_L_), medium QC1 (QC_M1_), medium QC2 (QC_M2_) and high QC (QC_H_) at concentrations of 10.00, 30.00, 1000.10, 2000.20 and 3000.29 ng/mL, respectively.

### 3.4. Sample Preparation

A total of 250.0 μL of plasma samples and 50.0 μL of IS (3000.50 ng/mL) were measured into a 2 mL microcentrifuge tube. The sample was vortexed for 30 s and centrifuged at 10,400 × *g* for 5 min after adding 1.0 mL of methanol. PTFE membrane was used to filter the supernatant (0.20 μm, 25 mm filter, Xiboshi Tianjin, Tianjin, China) prior to injection into the system.

### 3.5. System Suitability

Five replicates of 3250.32 ng/mL system suitability plasma samples, a solvent with a composition of water and methanol (1:1, *v/v*) and LLOQ were used. System suitability was performed at the beginning, during and end of the run to evaluate the drift and carry over of the system for method validation runs [36,37].

### 3.6. Method Validation

The validation parameters, namely specificity, sensitivity, linearity, carry over, matrix effect, precision, accuracy, dilution integrity, re-injection reproducibility and stability were performed according to the EMA Guidelines on Bioanalytical Method Validation [38] and recovery was conducted according to the FDA Bioanalytical Method Validation Guidance for Industry [39].

Seven batches of blank human plasma and spiked human plasma at LLOQ level were used to evaluate the specificity to ensure that there were no interference peaks at the retention times of etoricoxib and IS. The sensitivity of the method was based on signal to noise (S/N) ratio at LLOQ level using seven batches of human plasma.

The linearity of etoricoxib was constructed by plotting the etoricoxib peak area/IS peak area of eight non-zero standards versus the nominal concentrations of etoricoxib. A least square linear regression with a weighting factor of 1/*x*^2^ was applied to all calibration curves generated during the study.

A processed blank human plasma sample was injected right after ULOQ (4000.39 ng/mL) in each run to evaluate the carry over of the method.

The within-run accuracy and precision were evaluated using six replicates of LLOQ and QC samples (LLOQ, QC_L_, QC_M1_, QC_M2_ and QC_H_), on the same day. Six replicates of LLOQ and QC samples were analyzed on three different days for evaluation of between-run precision and accuracy.

Extended batch-run precision and accuracy were determined using twenty-five replicates of four QC samples (without LLOQ samples) analyzed in a single run together with system suitability and plasma standard curve to ensure that the precision and accuracy of each QC sample was within the acceptable limits in a size ≥ analytical run of study samples.

Matrix effect was assessed by comparing the peak area ratio of etoricoxib in the presence of matrix (processed blank plasma spiked with etoricoxib and IS) with the peak area ratio in the neat solution of etoricoxib at QC_L_ and QC_H_ levels.

The recovery was assessed by comparing the pre- and post-spiked peak responses of etoricoxib and IS in human plasma using six replicates at LLOQ and QC samples. Dilution integrity was performed by spiking the human blank plasma with etoricoxib working standard solution at a concentration above ULOQ (4800.47 mg/mL) and diluted with blank human plasma at 2 folds (2400.24 ng/mL) and 10 folds (480.05 ng/mL).

QC samples (QC_L_, QC_M1_, QC_M2_ and QC_H_) of three replicates for each concentration and a set of plasma standard calibration curves were injected three times at different intervals and the obtained results were compared with initial results for re-injection reproducibility evaluation.

Three replicates of QC_L_ and QC_H_ samples were prepared to evaluate etoricoxib short-term stability for 24 h, post preparative stability (at room temperature and in autosampler) for 48 h, long-term stability in the freezer for 94 days and freeze–thaw stability for seven cycles. The stability of etoricoxib and IS stock and working standard solutions was evaluated in two storage conditions, namely in the chiller and at room temperature for 94 days.

### 3.7. Comparative Dissolution Profiles

Comparative dissolution profiles (CDP) were performed to compare the in vitro performance of the test and reference formulations in different dissolution media comprising phosphate buffers (pH 1.2, 4.5 and 6.8). The dissolution study was conducted using a PharmaTest dissolution system (Hainburg, Germany) Apparatus II (paddle method) at 50 rpm and temperature of 37.0 ± 0.5 °C. The samples were collected at time intervals of 5, 10, 15, 20, 25, 30, 45, 60, 90 and 120 min. Similarity factor (*f*_2_) was used to conclude that the two dissolution profiles of test and reference products were similar.

### 3.8. Application to Bioequivalence Study

The bioequivalence study of Torixib film-coated tablet (120 mg etoricoxib, Y.S.P Industries (M) Sdn. Bhd., Malaysia) and Arcoxia film-coated tablet (120 mg etoricoxib, Frosst Iberica, Madrid, Spain under fasting conditions was approved by the Medical Research and Ethics Committee (MREC), Malaysia. The blood samples were collected before dosing (0 h) and at 0.25, 0.50, 0.75, 1.0, 1.25, 2.5, 2.0, 3.0, 4.0, 6.0, 8.0, 12.0, 16.0, 24.0, 36.0, 48.0 and 72.0 h after administration of drug products. The blood samples were centrifuged and the plasma was transferred into a plain tube before storage in the freezer at −20 ± 10 °C until sample analysis.

A total of 88 out of 791 samples (11.13%) were chosen for an incurred sample re-analysis (ISR). A total of two points for each period at C_max_ and elimination phase were selected for ISR study. The concentrations of the selected points at the elimination phase within four times of the LLOQ level of the study were selected for the ISR study.

### 3.9. Pharmacokinetic Analysis and Statistical Analysis

Two pharmacokinetic parameters, C_max_ and T_max_, were obtained from plasma concentration versus time profiles. A linear up logarithmic down method was used to calculate the area under the plasma concentration–time curve (AUC). Elimination constant (k_e_), elimination half-life (t_1/2_) and mean residence time (MRT) were calculated.

The ln-transformed data analysis of C_max_ and AUC_0–72_ of the two formulations was carried out using SAS software (version 9.3, Cary, North Carolina, USA). An analysis of variance (ANOVA) was performed to distinguish effects due to sequence, formulation, period and subject within sequence [40,41]. Bioequivalence was confirmed when the 90% confidence interval (CI) of the geometric mean ratios of the two main pharmacokinetic parameters (C_max_ and AUC_0–72_) of test/reference formulations was within 0.80–1.25 (80.00–125.00%).

## 4. Conclusions

The HPLC-ESI-MS/MS method for measurement of etoricoxib in human plasma was validated and applied to a bioequivalence study of two etoricoxib formulations. Methanol was used as protein precipitation agent in sample preparation and proved to be robust and simple. The present method was sensitive (S/N > 40 for etoricoxib), economical (one-step methanol protein precipitation technique), selective (K prime > 2), utilized small injection volume (1 μL) and short run time (2 min), and had negligible matrix effect and high recovery value. The ISR indicated that the method was highly robust and reliable. Etoricoxib exhibited secondary peaks after oral administration of 120 mg dose. Both formulations were bioequivalent and can be used interchangeably.

## Figures and Tables

**Table 1:** Summary of published bioanalytical methods for the quantification of etoricoxib in biological matrices.

References	Analytical Method	Type of Biological Matrix	Calibration Range	Sample Reparation Method	Injection Volume (μL)	Analysis Time (min)	Recovery (%)
[20]	HPLC-UV	Human plasma	10–750 ng/mL	Acetonitrile PPT	20	NA	97.53–98.96
[21]	HPLC-UV	Human plasma	5–5002.9 ng/mL	LLE	50	10	NA
[22]	HPLC-UV	Human plasma	20–2500 ng/mL	LLE	20	15	79.53–85.70
[23]	HPLC-UV	Human plasma	5–2500 ng/mL	LLE	100	10	75.60–76.60
[24]	HPLC-UV	Human plasma	15–3200 ng/mL	LLE	100	10	76.50–80.50
[25]	HPLC-UV	Human plasma	0.1–50 μg/mL	LLE	100	45	83.00
[26]	LC-MS/MS	Human plasma	0.2–200 ng/mL	SPE	15	2.5	>90.00
[27]	LC-MS/MS	Human plasma	1–5000 ng/mL	SPE	20	2	93.72–96.18
[28]	LC-MS/MS	Human plasma	10–2500 ng/mL	LLE	20	10	104.50
[29]	LC-MS/MS	Human plasma	5–5000 ng/mL	Acetonitrile PPT	3	2.5	94.25–96.48
[30]	LC-MS/MS	Human plasma	1–5000 ng/mL	LLE	20	2	92.74–98.32
[31]	HPLC-UV	Human plasma	30–3000 ng/mL	LLE	NA	>8.6	96.56–102.57
Present method	LC-MS/MS	Human plasma	10–4000.39 ng/mL	Methanol PPT	1	2	91.86–95.27

HPLC: High performance liquid chromatography; LC-MS/MS: Liquid chromatography tandem mass spectrometry; LLE: Liquid–liquid extraction; NA: Not available; PPT: Protein precipitation technique; SPE: Solid phase extraction; UV: Ultraviolet-visible.

**Table 2:** Within-, between- and extended batch-run precision and accuracy results.

Analyte		Etoricoxib Samples (ng/mL)				
		10.00	30.00	1000.10	2000.20	3000.29
Within-run 1 (*n* = 6)	Mean	10.85	30.94	1026.07	2050.84	2956.48
	CV (%)	16.28	7.16	1.33	1.22	1.12
	Bias (%)	5.15	3.14	2.60	2.53	−1.46
Within-run 2 (*n* = 6)	Mean	10.54	31.34	1052.11	2092.15	3018.59
	CV (%)	9.73	7.74	1.57	0.91	0.64
	Bias (%)	5.41	4.47	5.20	4.60	0.61
Within-run 3 (*n* = 6)	Mean	10.70	31.59	988.23	1983.04	2874.57
	CV (%)	16.67	5.92	0.80	0.88	1.08
	Bias (%)	7.04	5.29	−1.19	−0.86	−4.19
Between-run (*n* = 18)	Mean	10.59	31.29	1022.14	2042.01	2949.88
	CV (%)	13.74	6.61	2.90	2.46	2.25
	Bias (%)	5.86	4.30	2.20	2.09	−1.68
Extended-run (*n* = 25)	Mean	-	27.37	996.39	1949.15	2879.18
	CV (%)	-	6.75	4.97	4.53	3.87
	Bias (%)	-	−8.77	−0.37	−2.55	−4.04

CV: Coefficient of variation, *n*: Number of replicates.

**Table 3:** Recovery results of etoricoxib and etoricoxib D4 IS (*n* = 6).

Analyte	Nominal Concentration (ng/mL)	Mean ± SD (%)	Precision (CV, %)
Etoricoxib	30.00 (QC_L_)	95.27 ± 7.36	7.73
	1000.10 (QC_M1_)	91.86 ± 0.55	0.60
	2000.20 (QC_M2_)	94.03 ± 1.50	1.60
	3000.29 (QC_H_)	94.73 ± 1.01	1.07
Etoricoxib D4 (IS)	3000.50	93.76 ± 0.96	1.02

CV: Coefficient of variation, *n*: Number of replicates, SD: Standard deviation, IS: Internal Standard.

**Table 4:** Dilution integrity results of etoricoxib (*n* = 5).

Dilution Factor	Calculated Conc. (ng/mL) *	Mean ± SD (ng/mL)	Precision (CV, %)	Accuracy (Bias, %)
2-fold	4729.68	4811.62 ± 53.15	1.10	0.23
	4868.61			
	4835.08			
	4832.20			
	4792.52			
10-fold	5065.44	5054.65 ± 23.31	0.46	5.29
	5063.39			
	5082.05			
	5023.19			
	5039.18			

* After multiplying with dilution factor, CV: Coefficient of variation, Conc.: Concentration, *n*: Number of replicates.

**Table 5:** Stability study results of etoricoxib in human plasma, stock/working standard solution and IS stock/working standard solutions (*n* = 3).

Compound	Testing Conditions	Matrix	Concentration (ng/mL)	Bias (%)	Precision (CV, %)
Etoricoxib	Short-term (25 ± 4 °C), 24 h	Plasma	QC_L_, 30.00	2.96	5.27
			QC_H_, 3000.29	4.24	3.01
	Post-preparative in autosampler (25 ± 3 °C), 48 h		QC_L_, 30.00	−1.79	6.62
			QC_H_, 3000.29	−1.43	1.57
	Post-preparative at room temperature (25 ± 3 °C), 48 h		QC_L_, 30.00	2.76	5.10
			QC_H_, 3000.29	−1.31	1.40
	Freeze and thaw, 7 cycles		QC_L_, 30.00	−0.35	1.41
			QC_H_, 3000.29	1.42	2.98
	Long-term (−20 ± 10 °C), 94 days		QC_L_, 30.00	−0.06	2.91
			QC_H_, 3000.29	5.62	2.86
	Room temperature (25 ± 4 °C), 94 days	Stock standard solution	LLOQ, 10.00	0.18	2.69
			ULOQ, 4000.39	2.87	1.11
	Chiller (5 ± 3 °C), 94 days		LLOQ, 10.00	−4.93	5.15
			ULOQ, 4000.39	1.95	1.02
	Room temperature (25 ± 4 °C), 94 days	Working standard solution	LLOQ, 10.00	−6.18	2.48
			ULOQ, 4000.39	0.59	0.30
	Chiller (5 ± 3 °C), 94 days		LLOQ, 10.00	−4.48	0.36
			ULOQ, 4000.39	−1.49	1.15
Etoricoxib D4 (IS)	Room temperature (25 ± 4°C), 94 days	Stock standard solution	3000.29	1.81	0.25
	Chiller (5 ± 3 °C), 94 days		3000.29	2.70	0.46
	Room temperature (25 ± 4 °C), 94 days	Working standard solution	3000.29	2.03	0.56
	Chiller (5 ± 3 °C), 94 days		3000.29	2.31	0.40

CV: coefficient of variation, SD: standard deviation, *n*: number of replicates, LQC: low quality control, HQC: high quality control, IS: internal standard.

**Table 6:** Pharmacokinetic data of test and reference products in healthy volunteers after single dose of 120 mg of etoricoxib under fasting conditions (mean ± SD, *n* = 18).

Parameters (Unit)	Etoricoxib (Mean ± SD)	
	Test	Reference
C_max_ (ng/mL)	2220.27 ± 456.86	2233.90 ± 468.86
AUC_0–72_ (h.ng/mL)	38,656.80 ± 10,798.28	38,851.00 ± 11,123.90
T_max_ (h)	1.35 ± 0.55	1.41 ± 0.73
t_1/2_ (h)	24.93 ± 8.26	30.09 ± 15.15
k_e_ (1/h)	0.03 ± 0.01	0.03 ± 0.01
MRT (h)	34.05 ± 11.76	40.31 ± 20.29

SD: standard deviation, AUC: area under curve, MRT: mean residence time.

## Data Availability

The presented data in the study are available from the corresponding author upon request.

## Appendix A

**Table A1:** Optimized MRM parameter of etoricoxib and etoricoxib D4.

Analyte	Precursor Ion	Type of Ion Transition	MRM Transition (Product Ion)	Q1 Pre Bias (V)	Collison Energy (V)	Q3 Pre Bias (V)
Etoricoxib	359.15	Quantification	279.10	−18.0	−40.0	−30.0
		Confirmation	280.10	−18.0	−31.0	−30.0
Etoricoxib D4	363.10	Quantification	282.10	−19.0	−43.0	−30.0
		Confirmation	284.15	−19.0	−31.0	−21.0
